# EHMTI-0032. Persistence and switching characteristics among chronic migraine patient population: a retrospective claims analysis

**DOI:** 10.1186/1129-2377-15-S1-G8

**Published:** 2014-09-18

**Authors:** Z Hepp, D Dodick, S Varon, P Gillard, N Mathew, J Chia, R Hansen, EB Devine

**Affiliations:** 1 Global Health Outcomes Strategy and Research, Allergan Inc., Irvine, USA; 2 Neurology, Mayo Clinic, Phoenix, USA; 3 Global Safety and Epidemiology, Allergan Inc., Irvine, USA; 4 Pharmaceutical Outcomes Research and Policy, University of Washington, Seattle, USA

## Objective

Migraine prevention guidelines recommend oral migraine preventive medications (OMPMs) for patients with frequent headache, such as chronic migraine (CM). We sought to understand OMPM treatment patterns by evaluating persistence, medication switching, and medication re-initiation among CM pa

## Methods

A retrospective claims analysis was undertaken using the MarketScan® Databases. The analysis included patients ≥18 years old, with a CM diagnosis, who initiated an OMPM between January 1, 2008 and September 30, 2012. Patient persistence was measured at 6 and 12 months. Time to discontinuation was assessed for each OMPM and compared using Cox regression models. Among those who discontinued, we also assessed the proportion of patients who switched to another OMPM within 60 days or re-initiated treatment with OMPMs between 61 to 365 days.

## Results

A total of 8,707 patients met the inclusion/exclusion criteria. Persistence with the initial OMPM was 25% at 6 months and 14% at 12 months. Amitriptyline, nortriptyline, and gabapentin had significantly higher likelihood of discontinuation when compared to topiramate. Of patients who discontinued, approximately 23% switched to another prophylactic and another 41% reinitiated therapy within one year. Among patients who switched, 12-month persistence was 13% to the second prophylactic and 10% for the third. Among reinitiated patients this rate was 8% and 4%, respectively.

## Conclusion

Persistence to OMPMs is poor among the US CM population at 6 months and declines further by 12 months. After initial discontinuation, switching or re-initiating is common; however, persistence declines as patients cycle through various OMPMs.

